# Incomplete Kawasaki Disease in an Infant: A Case Report and Literature Review

**DOI:** 10.7759/cureus.22122

**Published:** 2022-02-11

**Authors:** Isra Idris, Abdalaziz M Awadelkarim, Eltaib Saad, John Dayco, Susan Beker

**Affiliations:** 1 Pediatrics, Woodhull Medical Center, New York City, USA; 2 Internal Medicine, Wayne State University Detroit Medical Center, Detroit, USA; 3 Internal Medicine, AMITA Saint Francis Hospital, Evanston, USA; 4 Pediatric Cardiology, New York University, New York, USA

**Keywords:** incomplete kawasaki disease in infants, non-fever kawasaki, coronary artery aneurysm, incomplete kawasaki disease, kawasaki disease

## Abstract

The term "incomplete Kawasaki Disease (IKD)" was first used to describe patients with coronary complications who did not fulfill the classical diagnostic criteria for Kawasaki Disease (KD). The risk of coronary artery involvement is similar if not greater in cases of IKD. However, the recognition of IKD is challenging and often delayed, especially in infants. Multiple algorithms have been formulated to identify cases of IKD utilizing supplemental clinical, echocardiographic, and laboratory features. Although fever is not required for a diagnosis of KD in the Japanese guideline, most of the current guidelines, including those of the American Heart Association (AHA), consider the presence of fever for at least seven days a requirement for the diagnosis of both KD and IKD in infants.

We present a case of IKD in a four-month-old female who presented with fever for less than three days and did not follow the current AHA algorithm for IKD. An echocardiogram obtained 10 days later revealed a coronary artery aneurysm, and a retrospective diagnosis of IKD was made. A review of the literature identified similar cases with a growing consensus on the need to redefine the role of fever. Pediatricians should search for coronary artery lesions in cases of high clinical suspicion, even if the fever period is short, particularly in those less than six months. Additionally, further innovative research is directly needed to identify immunological and cellular markers that could be tested early in the course of the disease and guide the management.

## Introduction

Kawasaki Disease (KD) is an acute, self-limited systemic vasculitis of small and medium vessels and occurs mainly between six months and five years of age. It was named after Tomisaku Kawasaki, a Japanese pediatrician who described this febrile vasculitis for the first time in 1967. Coronary artery complications occur in 25% of affected children. Currently, it is recognized as the major cause of acquired heart disease in developed countries [1].

The term "incomplete Kawasaki disease (IKD)" was at first used to describe patients with coronary complications who do not fulfill the full diagnostic criteria for KD. Multiple studies have shown that the risk of coronary artery involvement is similar if not greater in cases of IKD. However, the recognition of IKD is challenging and often delayed [2-3]. Infants less than six months represent a special age group, as they are more likely to lack classical manifestations and have a higher risk for coronary artery involvement [4]. Multiple algorithms have been formulated to identify cases of IKD utilizing supplemental clinical, echocardiographic, and laboratory features. Although fever is not required for a diagnosis of KD in the Japanese guideline, the majority of current guidelines, including the American Heart Association (AHA), consider the presence of fever for at least seven days a requirement for the diagnosis of both KD and IKD infants [5]. We present a case of Incomplete Kawasaki disease (IKD) in a four-month-old female infant who presented with fever for less than three days and did not follow the current AHA algorithm for IKD.

## Case presentation

A previously healthy four-month-old Hispanic female presented to the emergency department (ED) with a fever of (102.1°F) and decreased oral intake of one-day duration. Physical examination was benign. Urine culture, chest X-ray, COVID-19, and influenza A&B polymerase chain reactions (PCRs) were all negative. She was discharged home on oral paracetamol. One day later (the second day of illness), she presented again to our ED with unremitting fever, irritability, bilateral eye redness, and rash in the torso. Physical examination was remarkable for fever 102.2°F, tachycardia, bilateral nonexudative conjunctivitis, erythematous cracked lips, and a polymorphic maculopapular rash covering her torso and extremities. Laboratory workup was significant for mild thrombocytosis (448,000/mm^3^), an elevation of acute phase reactants (c-reactive protein (CRP) 63 mg/L, erythrocyte sedimentation rate (ESR) 90 mm/h), procalcitonin 0.49 ng/dl, positive respiratory viral panel for Rhino/Enterovirus. Urine analysis, serum albumin, AST, and ALT were normal. Blood culture and urine showed no growth.

Screening for viral exanthem, including Adenovirus, RSV, parvovirus, measles, and EBV, was negative. Based on existent clinical and laboratory findings, the patient was admitted and was initially treated as a viral illness with intravenous hydration and antipyretics. The patient had a good response to supportive measures: fever subsided on the day of admission, inflammatory markers trended down, and the patient was discharged on day four of hospitalization (sixth day of illness). The patient was evaluated by cardiology and didn't fulfill the clinical or laboratory criteria for IKD. However, it was still considered as a differential diagnosis, and an echocardiogram was scheduled for outpatient follow-up.

**Figure 1:**
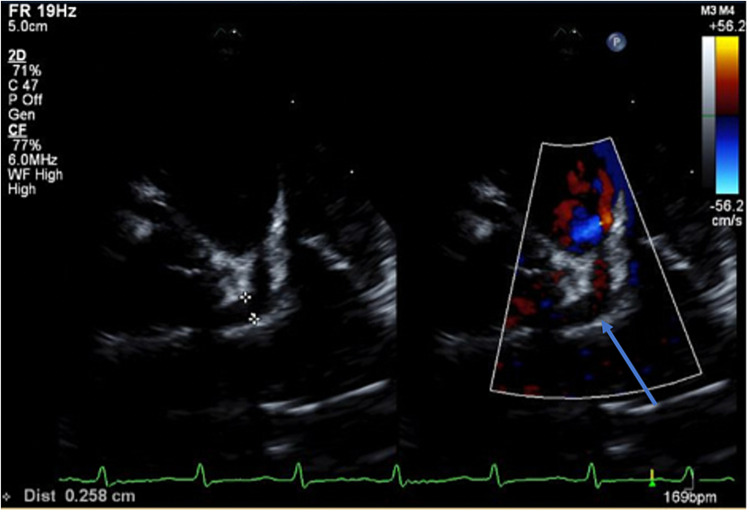
Transthoracic echocardiogram obtained on day 10 of illness showing a significantly dilated left main coronary artery (LMCA) (Z score = 3.4) (blue arrow)

On clinic follow-up three days after discharge (ninth day of illness), she had a persistent red tongue and lips; additionally, new swelling of both hands and feet was noticed. Her skin rash and conjunctival injection had resolved. The repeat platelet count was 750,000/mm. The EKG was normal. Echocardiogram showed normal ventricular function, significantly dilated left main coronary artery (LMCA) (Z score = 3.4), mild dilatation of the right coronary artery (RCA) (Z score = 2.5) and left coronary artery (LAD) (Z score = 2.6) (Figure 1).

**Figure 2:**
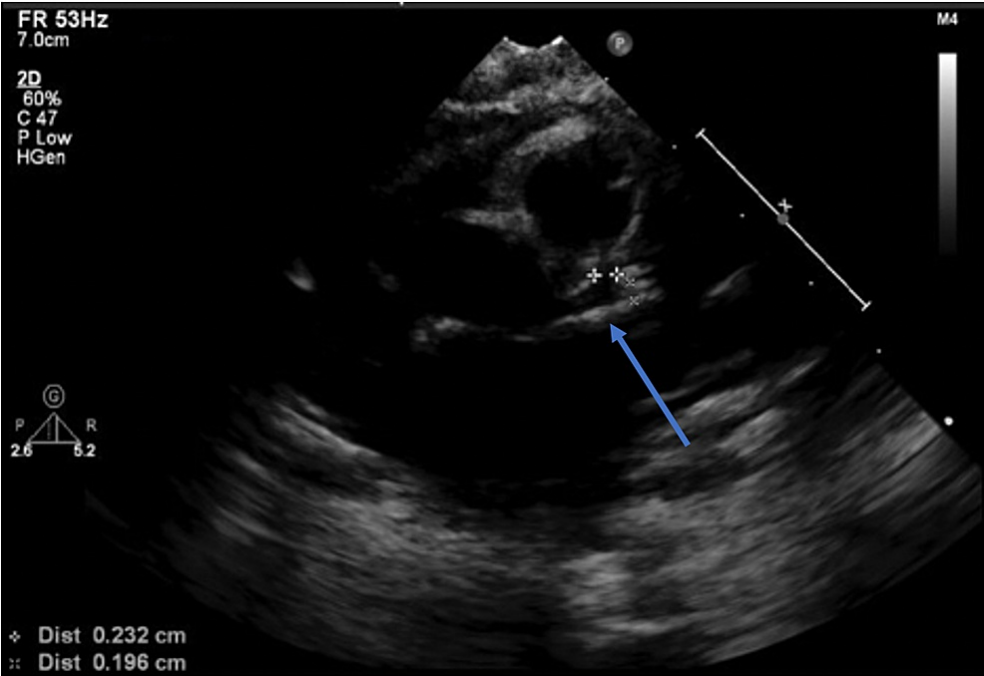
Transthoracic echocardiogram obtained at three months of illness showing resolution of left main coronary artery dilatation (blue arrow); left coronary artery ostium marked with plus markers.

In combination with clinical history and echocardiogram findings, she was readmitted and received intravenous immunoglobulin (IVIG), steroids, and aspirin. The patient had an uncomplicated hospital stay and was discharged three days later. A follow-up echocardiogram obtained six weeks into the illness showed resolution of coronary artery abnormalities (Figure 2). She remains in good health, and no further coronary artery abnormalities were identified on a three-month follow-up echocardiogram. The patient remained afebrile throughout her follow-up visits.

## Discussion

Complete KD occurs predominantly in the age group of six months to five years. The diagnostic criteria include fever for more than five days along with at least four of the following clinical features: erythema of the lips and oral mucosa, bilateral nonexudative conjunctival injection, polymorphous skin rash, changes in the extremities, and unilateral painless cervical lymphadenopathy. In the presence of ≥4 principal clinical criteria, mainly when redness and swelling of the hands and feet are present, the diagnosis may be made with only four days of fever. Typically, the clinical features are not all present at a single point in time, and it is generally challenging to establish the diagnosis very early in the course. If coronary artery abnormalities are detected, the diagnosis of KD is considered confirmed in most cases [5-6]. Echocardiography should be obtained at the time of diagnosis, one to two weeks later, and six weeks post-discharge. CT, MR, or conventional angiography should be considered for further risk stratification and complete coronary assessment in children who have significant coronary artery aneurysms on echocardiogram [6].

However, pediatricians sometimes encounter febrile children who do not meet the complete clinical picture but have several findings compatible with those of KD. In this situation, the identification of IKD is a clinical challenge, which can lead to delays in diagnosis and management. Echocardiographic changes take time to develop, and they are usually identified after the first week of illness [5]. It is well-established that the prompt recognition of KD and early initiation of IVIG, specifically within seven days, can decrease the incidence of coronary artery aneurysms from 25% to 4% [7-8]. Multiple studies have shown that the risk of coronary artery involvement is similar, if not greater, in cases of IKD [3]. Therefore, the current diagnostic criteria for KD are insensitive indicators for having or developing coronary complications.

A significant proportion of the burden of the disease occurs in infants less than six months of age (10%) [9]. This age group tends to have fewer typical clinical manifestations and has a higher prevalence of incomplete and atypical KD (40%) compared to older patients (10-12%) [10-11]. Fever and excessive irritability may be the only clinical manifestations of KD in infants. The presence of fever and pyuria in an infant can be mistakenly attributed to a urinary tract infection. Due to the elusive nature of the presentation, it's easy to understand why the diagnosis, and subsequently the treatment, of KD gets delayed in the infant group. As a result, they are usually diagnosed late and have a higher risk of coronary artery abnormalities, coronary artery aneurysms, and cardiac complications, including giant coronary artery aneurysms, shock, and death. Morbidity and mortality in this age group are highest compared to any other age group [6,12-13].

Park et al. compared patients with KD younger and older than six months and reported a higher incidence of coronary artery abnormalities (21.0% vs. 18.7%) and coronary artery aneurysms (4.7% vs. 3.1%) among the younger group [14]. Mastrangelo et al., in their recent single-center cohort of 113 patients younger than one year with KD, reported that infants with incomplete KD seem to have more severe disease and a greater predilection for coronary involvement compared to infants with complete KD [13].

The most recent algorithm of IKD by the AHA for infants includes ≥7 days of unexplained fever plus either three or more supplemental laboratory findings or typical echocardiographic findings (Z-score of the left anterior coronary artery (LAD) or right coronary artery (RCA) is ≥2.5). Additionally, the diagnosis may be considered in the following situations in infants less than six months: (1) prolonged fever and irritability; (2) prolonged fever and unexplained aseptic meningitis; (3) prolonged fever and unexplained or culture-negative shock; (4) prolonged fever and cervical lymphadenitis unresponsive to antibiotic therapy; (5) prolonged fever and retropharyngeal or parapharyngeal phlegmon unresponsive to antibiotic therapy [5].

The duration of fever has been confirmed as an important risk factor of coronary artery abnormalities [6,15]. There is a paucity of large-scale studies exclusively on KD below the age of 12 months regarding this topic. Non-fever KD has been reported in the available literature; nevertheless, it remains poorly defined with no clear consensus on its natural history and prognosis. From the scant reported cases, it appears to be more common in toddlers, and most cases had a mild course and few coronary artery abnormalities were identified [16-17]. In contrast, we were able to identify only two cases of infantile non-fever KD in the English literature. All reported cases, including our case, had significant echocardiographic findings reported [16,18].

The majority of existing guidelines consider the presence of fever for at least seven days a requirement for the diagnosis of both KD and IKD in infants. Therefore, it may induce a delay of management and postpone the diagnosis of IKD until the confirmation of coronary artery abnormalities, similar to our case. A decreased ability to mount a fever response may be present in some young infants, further contributing to the difficulty of diagnosing KD in this age group. Salgado et al., Singh et al., and Pilania et al. have all expressed similar concerns [12,19-20]. We do believe that the role of fever and the duration required to fulfill the criteria (especially in infants) needs to be redefined in the future.

There are many treatment options in the management of IKD, but no consensus has yet been established. The backbone of therapy includes IVIG and aspirin. Adjunctive therapies in the treatment included corticosteroids and biologic agents [5-6]. In our case, the patient was treated with IVIG as soon as the diagnosis of IKD was made; the patient had an excellent response with reduction of coronary artery dilatation and absence of new aneurysms on follow-up echocardiogram two weeks later. This case highlights that the diagnosis of IKD should be considered in infants presenting with unexplained fever even in the absence of the principal clinical findings of KD, particularly in those less than six months.

## Conclusions

Presently, the diagnosis of incomplete Kawasaki Disease might be made in cases with fewer classical diagnostic criteria and with several compatible clinical, laboratory, or echocardiographic findings. However, the diagnosis of IKD in infants less than six months of age remains a clinical challenge. This results in an increased rate of delayed diagnosis and subsequent risk of developing cardiac complications among this age group. Pediatricians should search for coronary artery lesions in cases of high clinical suspicion, even if the fever period is short, particularly in those less than six months. Additionally, further innovative research is very much needed to identify immunological and cellular markers that could be tested early in the course of the disease and guide management.
